# 

**Published:** 1842-09

**Authors:** 


					III.
SEPTEMBER, 1818.
:
No. L
?? ?
THE
A M E R ! CI N
?,*'
RARY
OF
?1 c n t a I 0 c i nt c e.
PUBLISHED UNDER THE AUSPICES OF THE
?QD ARVSRILV.
mrntvituu So|Cets ot Senttrt Stttraeotia.
editors:
CHAPIN a; HARRIS, M. D.
SOLYMAN BROWN, M. D.
LEONARD MACKALL, M. D
ADLAHD,
BALTIMORE:?ARMSTRONG &. BERBT,
HIGHLEY.
WOODS AND CRANE, *R8.5 BALT-
9 5 OO per annum, payable in adrance.

				

